# Retinoic Acid-Dependent Signaling Pathways and Lineage Events in the Developing Mouse Spinal Cord

**DOI:** 10.1371/journal.pone.0032447

**Published:** 2012-03-02

**Authors:** Marie Paschaki, Song-Chang Lin, Rebecca Lee Yean Wong, Richard H. Finnell, Pascal Dollé, Karen Niederreither

**Affiliations:** 1 Development and Stem Cells Department, Institut de Génétique et de Biologie Moléculaire et Cellulaire, Centre National de la Recherche Scientifique (UMR 7104), Institut National de la Santé et de la Recherche Médicale (U 964), Université de Strasbourg, Illkirch-Strasbourg, France; 2 Department of Medicine, Baylor College of Medicine, Houston, Texas, United States of America; 3 Center for Environmental and Genetic Medicine, Institute of Biosciences and Technology, The Texas A&M University System Health Science Center, Houston, Texas, United States of America; 4 Department of Nutritional Sciences, Dell Pediatric Research Institute, University of Texas, Austin, Texas, United States of America; 5 Department of Molecular and Cellular Biology, Baylor College of Medicine, Houston, Texas, United States of America; Baylor College of Medicine, United States of America

## Abstract

Studies in avian models have demonstrated an involvement of retinoid signaling in early neural tube patterning. The roles of this signaling pathway at later stages of spinal cord development are only partly characterized. Here we use *Raldh2*-null mouse mutants rescued from early embryonic lethality to study the consequences of lack of endogenous retinoic acid (RA) in the differentiating spinal cord. Mid-gestation RA deficiency produces prominent structural and molecular deficiencies in dorsal regions of the spinal cord. While targets of Wnt signaling in the dorsal neuronal lineage are unaltered, reductions in Fibroblast Growth Factor (FGF) and Notch signaling are clearly observed. We further provide evidence that endogenous RA is capable of driving stem cell differentiation. *Raldh2* deficiency results in a decreased number of spinal cord derived neurospheres, which exhibit a reduced differentiation potential. *Raldh2*-null neurospheres have a decreased number of cells expressing the neuronal marker β-III-tubulin, while the nestin-positive cell population is increased. Hence, in vivo retinoid deficiency impaired neural stem cell growth. We propose that RA has separable functions in the developing spinal cord to (i) maintain high levels of FGF and Notch signaling and (ii) drive stem cell differentiation, thus restricting both the numbers and the pluripotent character of neural stem cells.

## Introduction

Spinal cord development and neurogenesis are extremely interesting not only in the context of spinal injury and regeneration, but also in terms of the basic developmental processes that control cell lineage and specification. Complex molecular mechanisms guide progenitors cells in a specific spatial order that depend on their location according to the embryonic anteroposterior (AP) and dorsoventral (DV) axes. How patterning signals determine the identity of neural progenitors in a precise spatio-temporal order is still an issue of extensive study. Amongst the molecular pathways involved in this process is the retinoic acid (RA) pathway, which has multiple stage-specific functions in the generation, patterning, and maintenance of neural tissues [1], [2]. Initially neuroepithelial progenitors reside in a caudal neural plate ‘stem zone’ in which fibroblast growth factors (FGFs) act permissively to allow neural stem cell expansion [3], [4], [5], and initial stages of neural specification [6]. At these early stages RA appears to coordinate the progressive maturation of spinal cord progenitors (reviewed in [7]). RA synthesized by retinaldehyde dehydrogenase 2 (RALDH2) in differentiating paraxial (presomitic and somitic) mesoderm, diffuses towards the neuroepithelium and differentiates progenitor motor neurons (pMN) as they emerge from the caudal stem cell zone. Inhibiting RA signaling expands the size of the progenitor zone, seen as an enlarged caudal *Fgf8* expression zone [8], [9]. Retinoid deficiency inhibits the initiation of pMN differentiation, affecting *Pax6*, *Olig2*, *Ngn2* and *Bhlhb5* expression [10], [11].

The next steps in dorsoventral pMN patterning require ventral Sonic hedgehog (Shh) and dorsal Wnts secretion (reviewed in [12], [13]). The morphogenic action of these main signals differentially induces ventral or dorsal transcriptional targets, leading to the establishment of neuronal subtypes, so neuronal circuits can form and function independent of their initial inducers (reviewed in [14], [15]. Retinoic acid is intricately involved in many steps in this process (reviewed in [2]). Data on vitamin A-deficient quail embryos indicate that RA signaling is required for expression of dorsal patterning genes, playing additional roles in pMN and interneuron specification [16]. Retinoid roles at later stages include guiding the induction and patterning of lateral motor column (LMC) neurons [17] by regulating AP patterning genes including Hox genes [18]. Viable, tissue-specific mutants for the RA-synthesizing enzyme RALDH2 exhibit a reduced population of Lim1-positive brachial motoneurons, mispositioned LMC Islet1-positive neurons, and disregulated *Hoxc8*, leading to inappropriate axonal projection of nerves innervating extensor muscles and forelimb paralysis defects [17].

We have investigated these potential functions of RA by analyzing the spinal cord of *Raldh2^−/−^* null mutants rescued from early embryonic lethality by transient maternal RA supplementation [19], [20]. We show that dorsal spinal cord growth deficits are not due to abnormal Wnt- or dorsal-specific progenitor transcript levels. Rather, RA-deficient spinal cords are characterized by reduced dorsal FGF signaling and impaired expression of several Notch effectors. As a consequence, RA-deficiency inhibits neuronal stem cell proliferation, impairing neurosphere growth, differentiation and radial glial expression. Cell sorting experiments further show an expansion of the ‘side population’ (SP) of putative stem cells in the retinoid-deficient spinal cord. According to their transcriptional profiles, these cells were diverted from differentiation towards radial glia and maintained as pluripotent precursors and/or neural stem cells. In addition, analysis of spinal cord-derived neurospheres indicates that RA promotes neuronal differentiation *versus* pluripotent precursor maintenance.

## Results

### Rescued *Raldh2^−/−^* mutants as a model for RA deficiency in the differentiating spinal cord

To analyze RA-dependent events in the differentiating mouse spinal cord, we took advantage of a rescue system allowing to postpone the lethality of the *Raldh2^−/−^* mutants (occurring at embryonic day E9.5). This can be achieved by providing RA at early developmental stages via the maternal food. The doses administered are non-teratogenic, but are sufficient to rescue early cardiovascular abnormalities in *Raldh2^−/−^* embryos, and to obtain mutants for analysis until E14.5 [21], [22]. The minimal time frame for such a rescue is a 24 hour administration from E7.5 to 8.5 (hereafter designated as ‘short-term’ RA supplementation). The RA supplementation can also be extended for several days, testing whether abnormalities in *Raldh2^−/−^* mutants might be rescued in a non-cell autonomous manner (see below).

**Figure 1:**
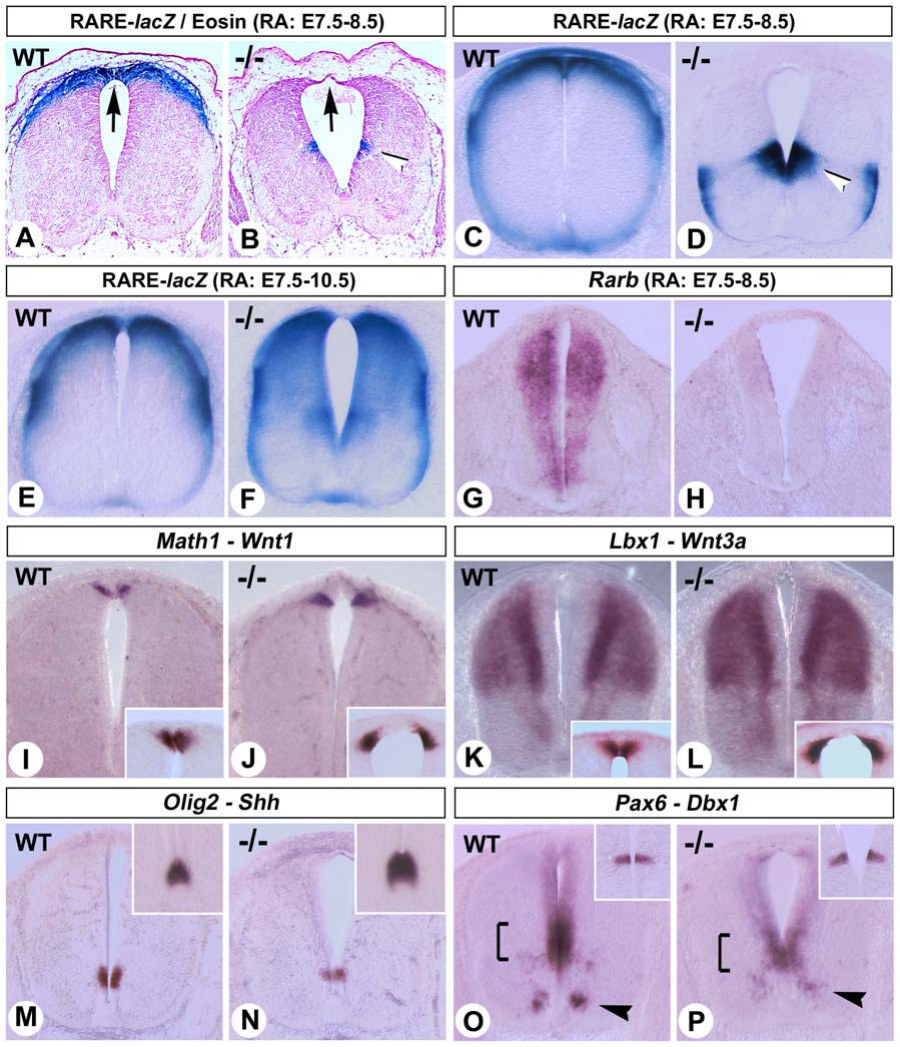
Short-term RA-rescue of *Raldh2^−/−^* embryos reveals abnormal dorsal spinal cord development. (A,B) Transverse paraffin sections of the brachial spinal cord of E12.5 WT (A) and *Raldh2^−/−^* (B) embryos harboring the RARE-*lacZ* reporter transgene, collected after short-term RA-rescue (E7.5–8.5). Embryos were X-gal stained prior to sectioning, and sections counterstained with eosin. Black arrows show defective roof plate development in the mutant. (C–F) Transverse vibratome sections of WT and *Raldh2^−/−^* embryos harboring the RARE-*lacZ* transgene, collected at E12.5 after short-term (C,D) or extended (E7.5–10.5; E,F) rescue, were processed for X-gal staining. These experiments confirmed the lack of RARE-*lacZ* activity in the dorsal spinal cord of short-term rescued mutants (B,D), whereas dorsally-restricted transgene activity was not properly restored upon extended rescue (E,F). A region of ectopic *lacZ* activity is seen in the middle region of the spinal cord ventricular layer, particularly under short-term rescue (white arrowheads in B,D). (G,H) ISH analysis of *Rarb* expression in WT and *Raldh2^−/−^* embryos collected at E11.5 after short-term RA-rescue (vibratome sections of the cervical/brachial spinal cord). (I–L) ISH analysis of *Wnt1* (I,J, insets), *Wnt3a* (K,L, insets), *Math1* (I,J, main panels), and *Lbx1* (K,L, main panels) in E12.5 WT and *Raldh2^−/−^* embryos after short-term rescue. Both *Wnts* are expressed in cells on each side of the dorsal midline, and the pattern observed in mutants (J,L) reveals that both sides of the neuroepithelium did not properly merge during neural tube closure. (M–P) ISH analysis of *Olig2* (M,N, main panels), *Shh* (M,N, insets), *Pax6* (O,P, main panels) and *Dbx1* (O,P, insets) in E12.5 WT and *Raldh2^−/−^* embryos after short-term rescue. Brackets and arrowheads point to abnormal downregulations in developing interneuron and motor neuron populations, respectively.

Mutants recovered at E12.5 (Fig. 1A,B) or E14.5 (data not shown) after short-term RA supplementation consistently showed an abnormal spinal cord. Although the neural tube had closed, it was reduced in thickness dorsally, and instead of a roof plate only a thin layer of cells was present at the dorsal midline (Fig. 1A,B, arrows). To assess if the dorsal spinal cord defects were linked to a lack of active RA signaling, we used mice harboring the RARE-hsp68-*lacZ* transgene [23], a sensitive reporter for endogenous RA activity (e.g. ref. [24]). This transgene is strongly expressed in the dorsal-most spinal cord cells in E12.5 WT embryos (Fig. 1A,C), mirroring a conserved promoter domain regulating *Raldh2* expression [25]. In *Raldh2^−/−^* mutants after short-term RA supplementation, the dorsal domain of RARE-*lacZ* activity was absent, correlating with the abnormal thinning of the neuroepithelium and absence of a roof plate structure (Fig. 1B, D). A novel region of RARE-*lacZ* activity appeared in prospective interneurons, as previously described (Fig. 1B,D, white arrowheads) [19], [22]. Extending the RA supplementation until E10.5 improved dorsal spinal cord morphology in *Raldh2^−/−^* mutants, leading to dorsal activation of the RARE-*lacZ* reporter (Fig. 1F), yet RARE-*lacZ* activity was not as sharply restricted as in WT littermates (Fig. 1E). To further establish that RALDH2 is required for the induction of endogenous RA-responsive genes, we analyzed *Rarb* transcripts (Fig. 1G,H). Indeed, these were not detected in spinal cords of short-term supplemented mutants (Fig. 1H).

The specification of distinct classes of neurons initially involves diffusible signals originating from dorsal (Wnt/BMP induced) and ventral (Shh induced) patterning centers. Graded signals from these two sites induce DV-restricted homeodomain and basic helix-loop-helix (bHLH) transcription factors expression. These transcriptional targets in the mitotic progenitor zone define the dorsoventral organization of spinal cord [26], [27]. Unaltered *Wnt1*, *Wnt3a*, *Bmp2* and *Bmp4* expression in the ectoderm and/or dorsal spinal cord neuroepithelium of E12.5 *Raldh2^−/−^* mutants after short-term RA supplementation (Fig. 1I–L, insert panels, and data not shown) indicated that these dorsal inductive signals are intact. Roof plate-derived signaling is required to induce dorsal neuronal subtypes (reviewed in [12]). Further analysis of *Raldh2^−/−^* mutants showed correct DV distributions of the transcription factors *Math1*, *Ngn1*, *Ngn2* and *Mash1* (Fig. 1 I,J, and data not shown) marking roof plate-dependent dl1-dl3 dorsal interneuron populations [28], [29], [30]. This confirmed intact dorsal signaling in RA-deficiency mutants. Roof plate-independent *Lbx1* expression in dl4-dl6 interneurons [31] was also observed in *Raldh2^−/−^* mutants (Fig. 1K,L). Our analysis thus shows that several molecular abnormalities observed at early stages of neural tube development in RA-deficient quail [5], [16] or mouse models [10], [32], no longer appear after mid-gestation in the rescued *Raldh2^−/−^* mutants. This might be because (i) maternally administered RA could induce early regulatory events rescuing, in particular, expression of dorsal determinants, and/or (ii) other regulatory inputs eventually allowed to induce these regional determinants.Ventral patterning via Shh also appeared unaffected in E12.5 short-term rescued *Raldh2^−/−^* mutants, although the most severely affected mutants showed a subtle dorsal *Shh* expansion (Fig. 1 M,N, insert panels). While RA has been shown to affect interneuron *Dbx1* and *Dbx2* expression [33], these were unaltered in the rescued *Raldh2^−/−^* mutants (Fig. 1O,P, inserts, and data not shown) probably due to interneuron RA production. Expression of *Olig2*, though, was reduced, potentially due to the requirement for FGF signaling in its induction (Fig. 1M,N, main panels) [34], [35], [36]. As suggested by their early RA-dependence (Fig. S1) [10], [37], *Ngn2* and *Pax6* require sustained RALDH2 activity to achieve normal levels of expression in motor neuron and interneuron populations (Fig. 1O,P, arrowheads and brackets, and data not shown).

### Reduced FGF signaling in the dorsal spinal cord of RA-deficient embryos

**Figure 2:**
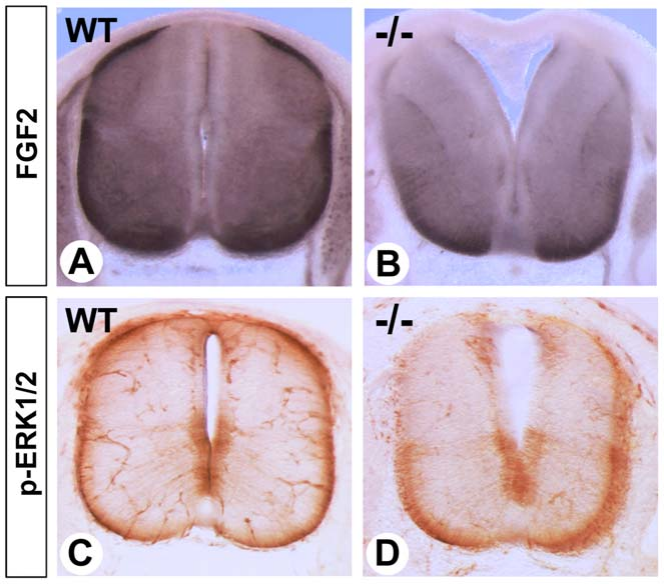
Decreased FGF activity in the spinal cord of RA-rescued *Raldh2^−/−^* mutants. Immunodetection of FGF2 (A,B, alkaline phosphatase staining) and p-ERK1/2 (C,D, peroxidase staining) on transverse vibratome section of the brachial spinal cord from E12.5 WT and *Raldh2^−/−^* embryos, collected after short-term rescue.

As RA regulates telencephalic growth [38] and neural specification by inducing FGF signaling [6], we examined whether deficiencies in this growth factor pathway might be responsible for retinoid-dependent dorsal spinal cord alterations. Immunolocalization of FGF2 revealed a striking absence in the most dorsal regions of the spinal cord in E12.5 mutants, whereas ventral levels were much less affected (Fig. 2A,B). Similar dorsal-specific reductions in pERK1/2 levels (Fig. 2C,D; Fig. S2A for western blot analysis)- indicative of intracellular FGF signaling [39]- revealed that the FGF pathway was compromised in the dorsal spinal cord. Forming spinal cord blood vessels also had strong pERK1/2 activity, again attenuated in the mutants (Fig. 2C,D). Extending RA supplementation until E10.5 restored both FGF2 and pERK expression in the dorsal spinal cord (data not shown).

These observations led us to investigate FGF signaling in the neural tube of unrescued *Raldh2^−/−^* embryos. While we confirmed that, at early somite stages, mutants display anteriorized *Fgf8* expression in the caudal neural plate [8], [9], from the 8 somite-stage onwards we observed reductions in FGF signaling, assayed by p-ERK immunolocalization, in the caudal neuroepithelium and paraxial mesoderm (Fig. S2B–E). The FGF-induced intracellular inhibitors *Sprouty1* (*Spry1*) and *Spry4* (Fig. S2F,G, and data not shown) and other *Fgfs* (*Fgf17* and *18*) were also reduced in mutants (Fig. S2H,I, and data not shown). Section analysis indicated decreased FGF2 expression in dorsal regions of the neural tube of unrescued E9.5 mutants, especially along the ventricular layer (Fig. S3A,B). p-ERK was also decreased along the ventricular layer in E9.5 mutants (Fig. S3C,D). At E10.5, in short-term rescued mutants, pERK immunoreactivity was concentrated in the ventral spinal cord, and was already attenuated in developing blood vessels (Fig. S3E,F).

### Transcriptomic analysis of the spinal cord

To further identify RA-dependent pathways regulating spinal cord formation, we performed transcriptome analysis of WT versus *Raldh2^−/−^* dissected brachial spinal cords collected at E12.5 after short-term RA rescue. Affymetrix DNA microarray analysis was performed on total RNA isolated from these samples. The validity of the screen was confirmed by reduced expression of known RA targets, such as *Rarb*, *Hoxb8* and *Hoxc6*, as well as *Raldh2* itself (Table 1). Analysis of transcriptional profiles using the EASE gene ontology clustering software revealed a striking number of retinoid-signaling components and homeobox transcription factors reduced in expression in the mutants (Table 1). These alterations were confirmed by ISH analysis of *Raldh2^−/−^* spinal cords (Fig. 1G,H, and data not shown). While reduced expression of selective structural proteins such as laminin α1 might disrupt basal lamina formation, an over-representation of TGFβ signaling and TGFβ target genes (such as procollagen I and VI [Table 1]) could contribute to the spinal cord structural defects in mutants. A similar hyperactivation of TGFβ signaling is observed in foregut tissues of *Raldh2^−/−^* mutants at E8.5, and has been implicated in the lack of lung induction [40] and cardiac outflow tract septation [41].

**Table 1:** Main categories of genes exhibiting reduced or increased expression levels from DNA microarray analysis of *Raldh2^−/−^* vs. wild-type E12.5 spinal cords (see Materials and Methods).

Retinoid Signaling - Reduced	Accession #	FC (E/B)	p value
retinol binding protein 1, cellular	NM_011254	0.392–16.0	0,013163
retinoic acid receptor, beta	BB266455	0.525–8.88	0,047686
aldehyde dehydrogenase 1 A2 (Raldh2)	NM_009022	0.540–5.43	0,041386
retinoic acid induced 2	BB770528	0.669–2.14	0,04899
SWI/SNF Smarca4	BG064918	0.883–1.3	0,02978

Homeobox Genes - Reduced			
hox C4	NM_013553	0.486–10.7	0,030597
hox B5	NM_008268	0.599–6.60	0,00246
hox A5	BC011063	0.624–5.66	0,03059
hox B6	BC016893	0.727–3.50	0,02776
hox B8	X13721	0.742–3.28	0,043881
homeodomain only protein	BC024546	0.807–2.46	0,008104
hox C6	BB440143	0.806–2.43	0,017862

Structural Proteins - Reduced			
laminin, alpha 1	NM_008480	0.777–2.79	0,01239
microtubule-associated protein 1 B	NM_008634	0.835–1.98	0,014958
amyloid beta binding protein (A) 2	BE952331	0.853–1.96	0,03334
neurofilament, light polypeptide	M20480	0.859–1.91	0,023978
collapsin response mediator protein 1	AB006714	0.877–1.76	0,014808
fascin-1	BE952057	0.877–1.77	0,016426

TGF-beta Induced - Increased			
transforming growth factor-β induced	BB533460	1.55+3.55	0,020205
procollagen, type I, alpha 2	BF227507	1.495+3.12	0,013626
connective tissue growth factor	NM_010217	1.45+2.8	0,008131
proline 4-hydroxylase	AI314028	1.32+2.09	0,030724
procollagen, type VI, alpha 2	BI455189	1.26+1.82	0,016774
transforming growth factor, beta 2	AW049938	1.19+1.55	0,043708
tenascin C	NM_011607	1.15+1.41	0,036012

Genebank accession numbers, Fold change (FC) ratios (Experimental versus Baseline, E/B) and Student's test p values are indicated.

### Reduced Notch signaling in the RA-deficient spinal cord

**Figure 3:**
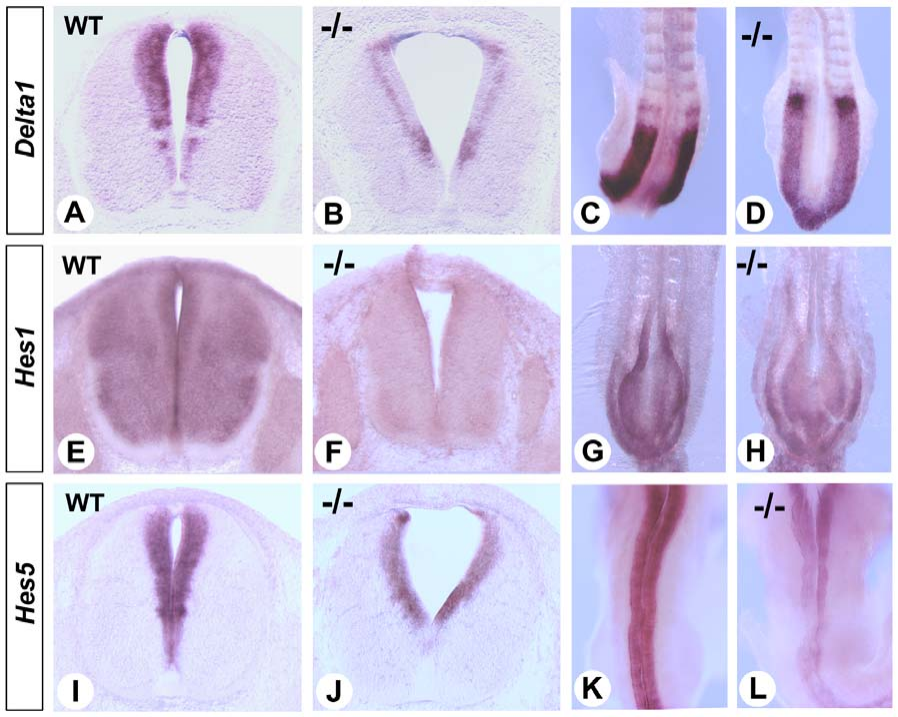
Decreased Notch signaling in the retinoid-deficient spinal cord. ISH analysis of *Delta1* (A–D), *Hes1* (E–H) and *Hes5* (I–L) was performed on transverse vibratome sections of brachial spinal cords from WT and *Raldh2^−/−^* embryos collected at E12.5 (A,B,E,F) or E11.5 (I,J) after short-term RA-rescue, and on whole-mount E8.5 unrescued embryos (C,D,G,H,K,L). Genotypes are indicated in each panel.

FGF signaling regulates uniform induction of Notch signaling as neuronal progenitors transition to an RA-dependent differentiation status [42]. While the Notch pathway has long been implicated in neuronal differentiation [43], [44], [45], recent studies point to important roles in neural stem cells selection and maintenance [46], [47], [48]. In mutants for the Notch effectors *Hes1* and *Hes5*, neuroepithelial cells are not properly maintained and radial glial cells prematurely differentiate into neurons [49]. In E12.5 *Raldh2^−/−^* spinal cords, *Delta1* transcripts (encoding a Notch ligand) were markedly reduced (Fig. 3A,B), whereas *Hes1* (Fig. 3E,F) and *Hes5* (Fig. 3I,J) expression were also affected. The same genes were downregulated in the early neural tube of unrescued *Raldh2^−/−^* mutants at E8.5 (Fig. 3C,D,G,H,K,L), consistent with observations made in avian models [5]. This further indicates that under RA deficiency, a failure to maintain FGF and Notch signaling might affect neuronal progenitor maintenance (see below).

### Disrupted dorsal root ganglia migration and survival

**Figure 4:**
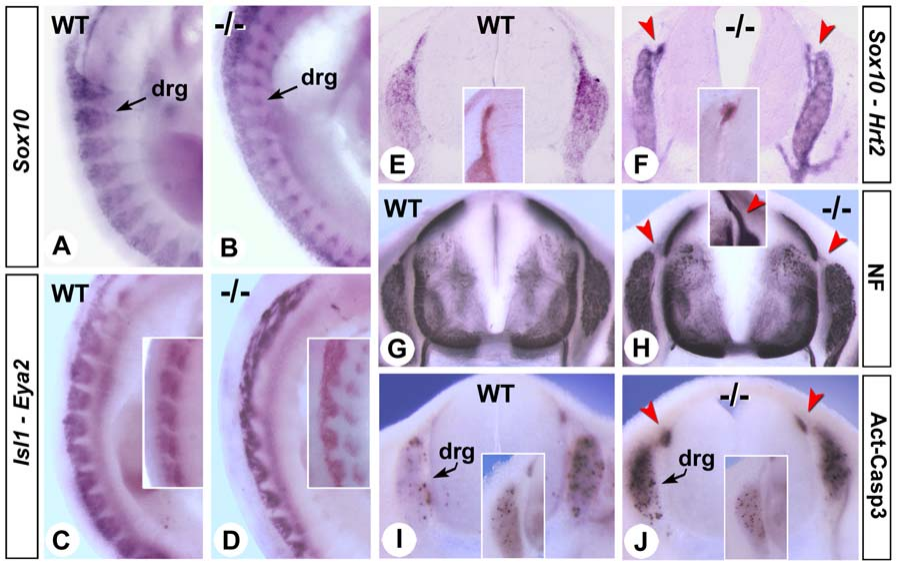
Retinoid deficiency affects dorsal root gangliogenesis. (A–D) Whole-mount ISH analysis of *Sox10* (A,B), *Isl1* (C,D, main panels) and *Eya2* (C,D insets) in the prospective dorsal root ganglia (drg) of E10.5 WT and *Raldh2^−/−^* embryos, collected after short-term rescue (genotypes as indicated). (E,J) ISH analysis of *Sox10* (E,F, main panels) and *Hrt2* (E,F, insets), anti-neurofilament (NF) staining (G,H) and activated-Caspase 3 immunodetection (I,J) on transverse vibratome sections of brachial spinal cords of E12.5 WT and *Raldh2^−/−^* embryos after short-term rescue (genotypes as indicated). Insets in H, I and J show details of a dorsal nerve root (H) and dorsal ganglia (I,J) of embryos collected at the same stage after an extended (E7.5–10.5) rescue. Red arrowheads indicate the dorsal nerve exit points, which are sites of excess apoptosis in the short-term rescued mutant (J).

Retinoids may control several steps of peripheral nervous system development, by triggering neural crest cell (NCC) epithelial-mesenchymal transition, coordinating dorsal root ganglion (DRG) formation along defined rostrocaudal pathways [50], and stimulating neurite outgrowth and sympathetic neuron survival [51], [52]. We examined trunk neural crest genesis and DRG formation in the rescued *Raldh2^−/−^* mutants. Unaltered expression of the neural crest markers *Sox10* and *Pax3* at E9.5 indicated no obvious defects on the generation of NCCs in short term RA-supplemented *Raldh2^−/−^* mutants (data not shown). However, postmigratory neural crest was disorganized at E10.5, as observed with several molecular markers including *Sox10*, *Isl1* and *Eya2* (Fig. 4A,C). In *Raldh2^−/−^* mutants, forelimb-level NCCs migrated as partly fused sheets rather than in segmental streams. Hence, developing DRG were not properly segregated, and gangliogenesis was compromised (Fig. 4B,D). Detailed analysis on sections of E12.5 mutants with *Sox10* (Fig. 4E,F), anti-neurofilaments (Fig. 4G,H), and the Notch ligand *Hrt2* (which marks the spinal nerves dorsal exit points, Fig. 4E,F, insets), showed that dorsal spinal nerves were disorganized and shortened, and their connections with the DRG were poorly defined (Fig. 4F,H, arrowheads).

During gestation many neurons (including some of the DRG) are eliminated by apoptosis due to a lack survival signals [53]. To examine if increased cell death accompanies DRG malformations, we performed immunodetection of the active form of caspase 3 (Act-Casp3) - a main effector of the apoptotic cascade (reviewed in [54]). Higher levels of Act-Casp3 labelling were observed in the DRG of *Raldh2^−/−^* mutants, and at the level of the dorsal spinal nerve exit points (Fig. 4J, arrowheads). This increase in cell death might account for the shortened dorsal nerve tracts and overall disorganization of the dorsal nerves and DRG in the mutants. These defects were prevented by extending the RA supplementation until E10.5: in that case the *Raldh2^−/−^* mutants showed similar numbers of Act-Casp3+ cells as controls (Fig. 4I,J, insets), and better organized dorsal nerve tracts (Fig. 4H, inset).

### Rescued *Raldh2^−/−^* mutants display an abnormal lateral motor column phenotype

**Figure 5:**
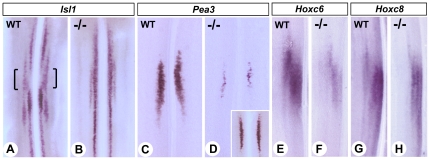
*Raldh2^−/−^* mutants exhibit severe reduction of developing brachial motor neuron pools. Whole-mount ISH analysis of *Isl1* (A,B), *Pea3* (C,D), *Hoxc6* (E,F) and *Hoxc8* (G,H) on dissected cervical/brachial regions of the spinal cords of WT and *Raldh2^−/−^* embryos, analyzed at E12.5 after short-term rescue (genotypes as indicated). All spinal cords are viewed as flat-mounts after cutting the dorsal midline, and only half-sides are shown in E-H. An inset in D shows the *Pea3* labelling observed in the brachial spinal cord of a *Raldh2^−/−^* embryos collected after an extended (E7.5–10.5) RA-rescue.

Spinal motor neurons arise from common ventral progenitor domains, and are organized in columns whose identities direct correct axonal projection [55], [56]. Retinoic acid generated by RALDH2 in postmitotic motor neurons acts to upregulate *Lim1* expression, which is required for lateral motor columnar (LMC_L_) subtype specification. This in turn regulates dorsal limb axonal projection pathways [17], [57]. Conditional gene inactivation strategies have revealed functional contributions of RALDH2 acting both within the paraxial mesoderm and in postmitotic neurons, in order to specify LMC_L_ identity [58], [59]. RA-supplemented *Raldh2^−/−^* mutants display severely disorganized brachial LMCs, as evidenced by the presence of continuous *Isl1*-expressing ventral columns, whereas these columns are interrupted by non *Isl1*-expressing cells in the WT spinal cord (Fig. 5A,B, brackets). These alterations are similar, or even more severe, than those described in LMC conditional *Raldh2* mutants. Furthermore, we found a severe reduction of the LMC motor pools marked by *Pea3* (Fig. 5C,D), *EphA4* (data not shown), *Hoxc6* and *Hoxc8* (Fig. 5E–H). This phenotype was partly rescued by extending the RA supplementation until E10.5, resulting in less severely reduced pools (Fig. 5D, inset). As each of the *Raldh2* conditional mutants [58], [59] displays a milder version of the LMC phenotype observed in the rescued *Raldh2^−/−^* null mutants, our data support the idea that diffusible RA produced mesodermally and RA generated by LMC neurons act synergistically to specify the LMC motor pools.

### Spinal cord neurosphere proliferation and survival is RA-dependent

**Figure 6:**
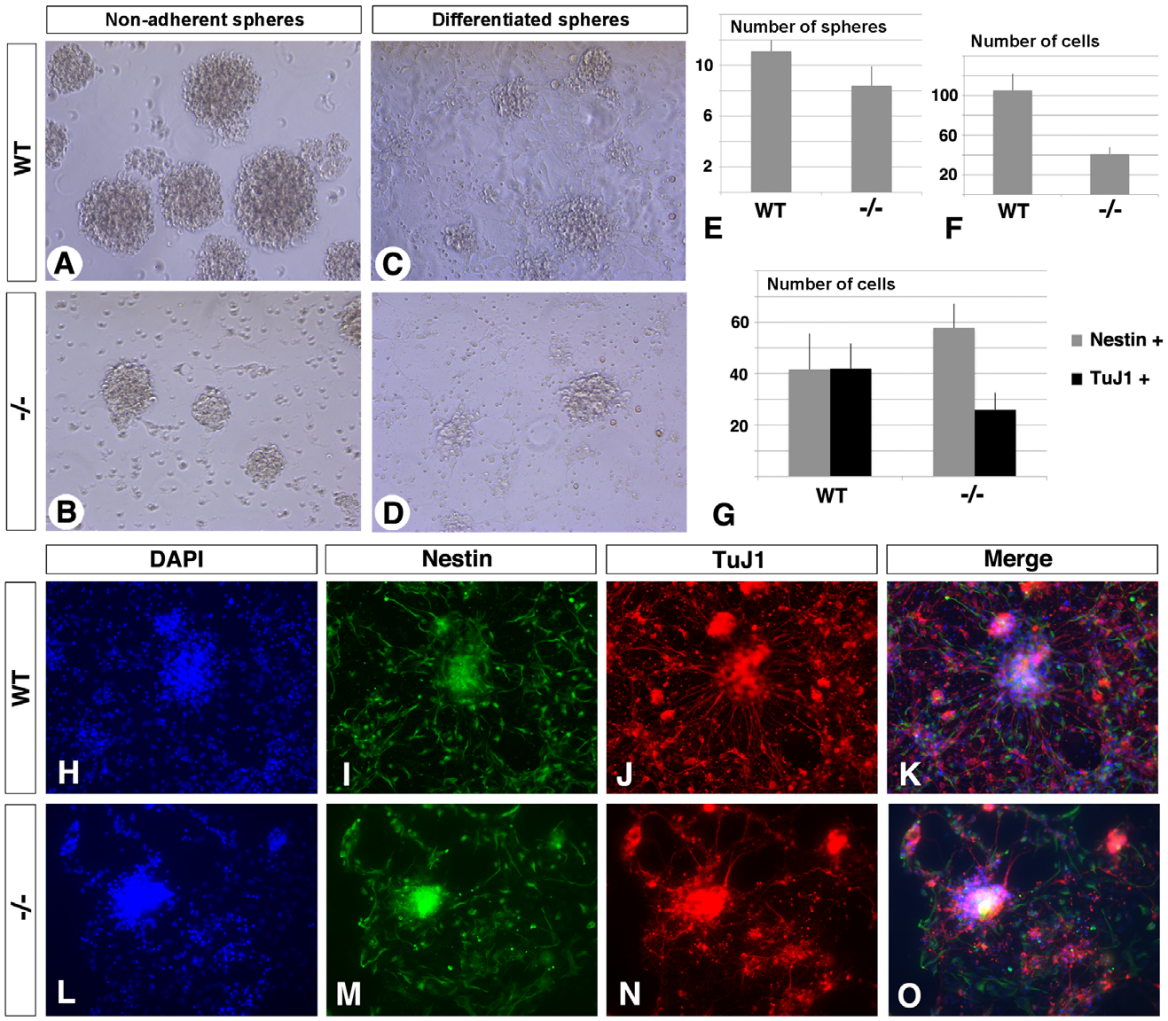
Abnormal development of neural stem cell-derived neurospheres from *Raldh2^−/−^* spinal cords. Neurospheres were derived from dorsal cervical/brachial spinal cord from E12.5 WT and *Raldh2^−/−^* embryos. (A,B) After growth of dissociated progenitor cells in suspension, in 6-well plates, for 10 days in a defined selective medium (see Materials and Methods), the number of spheres developing from *Raldh2^−/−^* embryos spinal cords is decreased by 25% compared to WT (E: 11.1±0.88 spheres/well for WT; 8.40±1.50 for mutants; n = 9; T- test: P value = 0,0009). (C,D) To further study progenitor cell differentiation, spheres were plated onto laminin-coated coverslips for 3 days, after 10 days of growth in suspension. *Raldh2^−/−^* derived spheres exhibited reduced number of cells compared to WT (F: one quadrant of a plated WT sphere is composed of 105.4±16.6 cells, against 40.9±6,82 cells in a *Raldh2^−/−^* sphere; DAPI positive nuclei were counted using ImageJ software; n = 9; P = 1.6×10^−5^). (G–O) After growth onto laminin coated coverslips for 3 days, cells were processed for immunocytochemistry. Nestin+ cells (I,M) are increased by 20% in the *Raldh2^−/−^*derived spheres, while TuJ1+ cells (J,N) are decreased by half. (H,L, DAPI staining; K,O, merged images). (G) After counting and normalization for total cell numbers, assessed by DAPI positive cell nuclei, one quadrant of a WT sphere contains 41.6±14 Nestin+ and 42.0±9.76 TuJ1+ cells, against 57.9±9.21 and 25.9±6,66 in a *Raldh2^−/−^* sphere (n = 9; P = 0,001 and 0,007, respectively).

As RA deficiency reduced FGF and Notch signaling, we hypothesized that spinal cord neural stem cells (NSCs) would be adversely affected. To directly test this hypothesis we examined whether RA deficiency affects the ability of spinal cord cells to form clonally derived neurospheres, using a floating serum-free assay where NSCs proliferate and generate multipotent clones [60]. Dorsal brachial/cervical spinal cord explants from short-term rescued E12.5 *Raldh2^−/−^* mutants were subjected to primary cultures, and progenitors enriched in the presence of FGF2 and EGF. Like their control littermates, *Raldh2^−/−^* spinal cords were able to generate neurospheres (Fig. 6A–D) after 8–12 days in culture. Interestingly *Raldh2^−/−^* spinal cord cultures exhibited a 25% reduction in neurosphere number at day 8 (Fig. 6E), and the resulting neurospheres were smaller in size when compared to WT controls (Fig. 6A–D).

Analysis of the ability of spinal cord-derived neurospheres to differentiate in vitro was performed to assess their lineage potential. Typically 10–12 spheres were allowed to attach to a laminin-coated coverslip. The absence FGF2 and EGF in culture stimulated this event. After 3 days, nestin positive neural progenitor cells and β-III tubulin (TuJ1 positive) differentiated neurons were detected in both WT and *Raldh2^−/−^* derived neurospheres. Notably, the nestin positive cell population was increased in the *Raldh2^−/−^* derived spheres by 20%, while differentiated neurons where decreased by half (n = 9) (Fig. 6G–O). This indicates that RA controls the fate of neuronal progenitors by promoting neuronal differentiation. The total number of cells in *Raldh2^−/−^* spheres was decreased by 50% in comparison to WT (Fig. 6F). The reduced fraction of differentiating neurons likely accounts for the progressively smaller size of mutant spheres under these conditions.

### RA deficiency increases the spinal cord side population (SP) and alters its transcriptional profile

**Figure 7:**
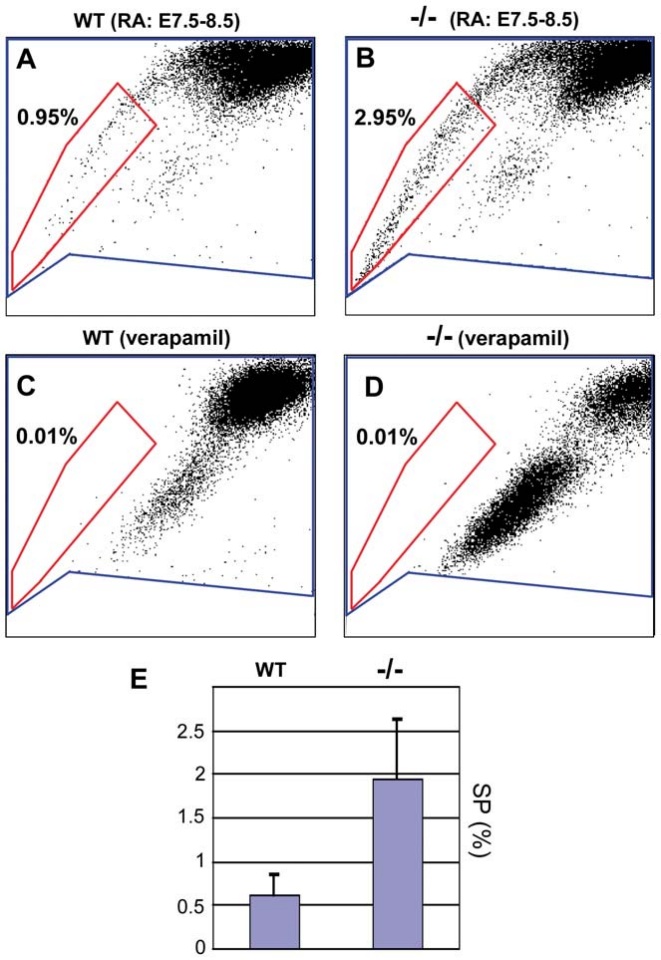
Retinoic acid deficiency increases the surrogate stem cell SP fraction in fetal spinal cord. A–D: Hoechst 33342 FACS profiles (see Materials and Methods) of cell suspensions from dissected spinal cords of E12.5 WT (A) and *Raldh2^−/−^* mutants (B), and from WT and mutant samples preincubated in the channel blocker verapamil prior to dye addition (C,D). Percentages of cells within the SP fraction (red) are indicated. E: Mean percentages of SP cells in WT and mutant E12.5 spinal cords, respectively (n = 9 cell-sorting experiments performed on independent pools of 10–20 WT or mutant samples, respectively).

Dual-wavelength flow cytometric analysis (FACS) of cells labeled with the fluorescent DNA-binding dye Hoechst 33342 can be used to assay for the differential ability of stem cells to efflux the Hoechst dye [61], [62]. Using this assay a small population of laterally shifted cells (the ‘side population’, SP) exhibits NSC potential and is inhibited in its terminal lineage commitment [63]. To investigate whether RA might regulate SP formation, brachial spinal cords were dissected at E12.5 from short-term rescued *Raldh2^−/−^* mutants and WT littermates, and assayed for SP cell numbers (n = 9 FACS experiments performed on independent pools of 10–20 spinal cords each). SP cells constituted approximately 0.6% of the sorted cell fractions from WT samples (Fig. 7A,E). Consistently, *Raldh2^−/−^* spinal cords exhibited a ∼3-fold increase in SP cells, with respect to WT samples (Fig. 7B,E). The Hoechst dye efflux capacity of stem cells is due to membrane efflux pumps of the ATP-binding cassette (ABC) transporter superfamily, including multidrug resistance 1 (MDR1) and ABCG2 [64], [65]. These pumps could be blocked by verapamil in both WT and mutants (Fig. 7C,D), showing the specificity of the assay.

**Figure 8:**
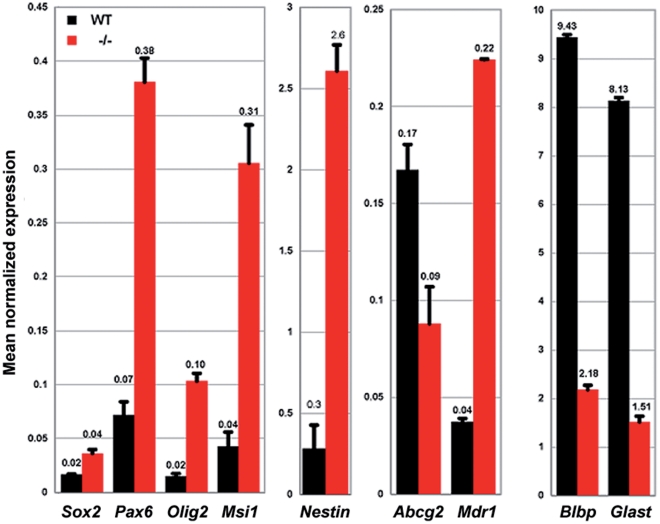
Retinoic acid deficiency alters gene expression profiles in spinal cord SP cells. Quantitative RT-PCR analysis of *Sox2*, *Pax6*, *Olig2*, *Msi1*, *Nestin*, *Abcg2*, *Mdr1*, *Blbp*, and *Glast* mRNA levels in SP fractions from WT and *Raldh2^−/−^* brachial spinal cords (black and red bars, respectively) collected at E14.5 after a short-term (E7.5–8.5) RA-rescue. Data are represented as relative mRNA levels with respect to *Gapdh* levels.

RNA was isolated from the sorted SP fractions, amplified and assayed by real-time quantitative PCR to test if the *Raldh2^−/−^* SP might be altered in its expression of pluripotency and/or NSC markers. Levels of the NSC markers *Msi1* and *Nestin* were upregulated by about 8–9 fold, data consistent with observations of increases in nestin positive cells in the neurosphere assays. Expression of the progenitor/neurogenic markers *Pax6*, *Olig2*, and *Sox2* was increased 2–6 fold in the mutant SP. The radial glia markers *Blbp* and *Glast*, though, were dramatically reduced under RA deficiency (Fig. 8). While levels of the *Mdr1* gene were increased 6-fold in the mutants, *Abcg2* levels were reduced by about 60% (Fig. 8). Collectively these changes indicate that the *Raldh2* mutant SP is shifted towards a more pluripotent/NSC transcriptional profile, but is inhibited in its ability to generate a radial glia lineage.

## Discussion

### Rescue of *Raldh2^−/−^* embryos reveals abnormal spinal cord development

*Raldh2* has a dynamic expression in the axial, paraxial and lateral plate mesoderm adjacent to the early embryonic neural tube. This is followed by specific expression in the developing meninges and lateral motor columns (LMC) of the spinal cord [17], [66]. Initially RA sets the expression boundaries of *Hoxc* genes [67], [68], then differentiates caudal FGF stem cell progenitors. Elaboration of DV programs of motor and interneuron differentiation [16], [33], [69] and induction of dorsal limb motor neuron subtypes also require RA [17], [57]. Our work has shown two levels of action of RA influencing spinal cord cells during development. RA acts globally (in both neuronal and non-neuronal lineages) to maintain high levels of FGF and Notch signaling. Additionally, it promotes neuronal progenitors differentiation. In the absence of RA, progenitor cells are maintained in a multipotent state; however, reduced FGF and Notch signaling will eventually lead to a depletion of neuronal progenitors and a block of differentiation toward the radial glia lineage.

### Neurite growth and cell survival is hindered in dorsal root ganglia of RA-deficiency mutants

In the peripheral nervous system RAR signaling appears to play a central role in regulating neurite extension (reviewed in [70], [71]). An upregulation of *Rarb* following RA treatment correlates with increased neurite outgrowth in cultured adult dorsal root ganglia (DRG), and lentiviral RARβ2 overexpression promotes regeneration of sensory axons, both in vitro and in vivo after cervical dorsal root crush injury [72], [73]. RA is likely to sustain axonal growth and neuronal survival by transcriptional activation of neurotrophins and their receptors [74]. Reciprocally, the neurotrophin NGF activates *Raldh2* transcription [75]. Through this regulatory loop, RA signaling will facilitate NGF and NT-3-dependent neurite outgrowth [51], [52], [76] and activate targets necessary for axonal elongation [77]. Upon *Raldh2* loss of function there is a clear reduction in the length of spinal nerves entering the DRG, which are predictably NGF and NT-3 RA-responsive populations. Actions of NGF increasing MAPK family signaling (SAPK and ERK1/2) and preventing DRG apoptosis [78], are presumably impaired in the dorsal spinal nerves of *Raldh2^−/−^* mutants. A similar function of RA in offsetting cell death has been shown in the hind- and forebrain [79], [80], potentially due to altered FGF signaling [38], [81] and neurotrophin availability [53].

### RA deficiency reduces FGF and Notch signaling, and neural stem cell survival

In spite of an increasing number of studies aiming to elucidate the mechanisms of regulation of neuronal subtype diversification, altogether spinal cord neurogenesis remains poorly understood. In vitro differentiation studies of embryonic stem (ES) cells are a powerful tool, offering access to molecular pathways driving neural stem cell differentiation (reviewed in refs. [82], [83]). Combinatorial interactions of trophic factors appear critical in inducing neural stem cells (NSCs) toward defined lineages. Interestingly, spatial-specificity can also be induced during NSC differentiation. As an example, motor neurons induced by combined RA and Shh action express markers of cervical level spinal motor neurons [83]. There is an intrinsic temporal patterning mechanism of ES cell neurogenesis, also occurring in the developing spinal cord. Neuron generation precedes that of glial cells. A late (E12.5+ in mouse) neuron-glial switch is a general property of NSCs in all parts of the developing brain and spinal cord. The Notch signaling pathway plays a pivotal role in the maintenance of stem/progenitor cells and the neuronal to glial switch in identity [84], [85], [86]. This Notch-regulated glial transition is controlled by combined action of nuclear receptors COUP-TFI and COUP-TFII (NR2F1, NR2F2) [87], the latter being found to be a RA-activatable receptor [88].

The effects of combined reductions in FGF2/p-ERK and Notch signaling in RA-deficient spinal cords might reflect more general functions of FGF2 in increasing neurons and glia generation from cortical stem cells [89], [90], and Notch signaling in first maintaining NSCs, then promoting glial fate transition [91]. Murine *Hes1*;*Hes5* double mutants have a premature depletion of radial glial cells, leading to structural alterations of the spinal cord and fusions of the DRG [92], reminiscent of the phenotype of the short-term rescued *Raldh2^−/−^* mutants. RA directs ES cells to become a uniform RC2+Pax6+ radial glial lineage [93], [94], indicating it may serve as a master regulator. Consistently, the fetal brain subventricular zone harbors subpopulations of RA-activated RC2+, GLAST+ radial glia progenitors [95]. RALDH2, by inducing meningeal or fibroblastic FGF2 production in both the fetal brain and spinal cord, may play a role in the stromal microenvironment allowing stem cell maintenance, consistent with reduced NSC survival under RA deficiency [96]; this study). The effects of RA on NSC populations appear synergistic, i.e. increasing both FGF and Notch signaling to promote NSC commitment [97].

### Perspectives for human health

We have shown that RA-deficiency abnormalities in spinal cord populations can be partly prevented by providing an extended maternal retinoid treatment, a finding which may be applicable in stem cell-based therapies. Recent studies in animal models clearly pointed out to a role of retinoids in promoting neurogenesis in the adult spinal cord [98], and stimulating axonal outgrowth after spinal injury [99]. More generally, in human RA deficiency might contribute to NSC degeneration and to some neurological diseases. Reductions in *Raldh2* expression and RA signaling have been found in postmortem samples of patients with human amyotrophic lateral sclerosis (ALS) and Alzheimer's disease [100], [101], both exhibiting neurodegenerative phenotypes. There is evidence for defective retinoid transport and function in late onset Alzheimer's disease [102]. One mechanistic role for RARα signaling in preventing amyloid-β accumulation at the onset of Alzheimer's disease is by increasing Notch related α-secretase activity via a direct induction of ADAM10, and Alzheimer's-like defects are indeed suppressed by RARα agonists in a transgenic mouse model [103]. Although the drug Memantine (an NMDA receptor antagonist) clinically used for treatment of Alzheimer's disease was found to increase the number of radial glial-like progenitor cells in adult mouse hippocampus [104], more generally the role of the radial glial lineage in neurodegenerative models is still debated.

Whether alterations in retinoid signaling during human embryonic and fetal development may have a causative role in neurogenesis defects and/or mental retardation (e.g. ref. [105]) is also unclear. Alcohol exposure appears to decrease embryonic RA levels [106], [107], potentially contributing to mental retardation associated with the fetal alcohol syndrome [108]. Exposure to ethanol during critical periods of development decreases the radial glial progenitor pool, and reduces levels of activated Notch1 and FGFR2 [109]. Impaired retinoid signaling secondary to overall vitamin A deficiency or fetal alcohol exposure, may in the long-term result in the selective depletion of radial glial NSCs. While RA treatment alone might partially restore these defects, understanding how synergistic combinations of RA, Notch, and FGF agonists boost endogenous neuroregenerative capacities may offer a plausible alternative to cell based treatments.

## Materials and Methods

### Ethics statement

All animals were maintained and manipulated under animal protocols reviewed by the Baylor College of Medicine and University of Texas at Austin ethics committees, which specifically approved this study (AUP 2010-00128), in strict accordance with NIH guidelines, provisions of the Guide for the Care and Use of Laboratory Animals, and the Animal Welfare Act.

### In situ hybridization (ISH) and immunohistochemistry (IHC)

*Raldh2*-null mutants, and the maternal RA supplementation procedure, have been described previously [20], [22]. All animals were maintained and manipulated under animal protocols reviewed and approved by the Baylor College of Medicine and University of Texas at Austin, in strict accordance with NIH guidelines, provisions of the Guide for the Care and Use of Laboratory Animals, and the Animal Welfare Act. Whole-mount ISH with digoxigenin-labeled riboprobes was performed as described [110], using Intavis InSituPro robots (for details, see http://www.empress.har.mrc.ac.uk/browser/, Gene Expression section). All ISH beyond E9.5 were performed on transverse (100 µM) vibratome sections of paraformaldehyde-fixed embryos. X-gal assays were performed as described [23]. Whole-mount immunolabeling with the phospho-p44/p42 Map-kinase (p-ERK1/2) antibody (Cell Signaling) or FGF2 (Santa Cruz) was performed according to the Rossant web site protocol (http://www.sickkids.ca/research/rossant/protocols/ImHis.asp). Anti-cleaved caspase-3 (Cell Signaling Technology) and mouse monoclonal anti-neurofilament (2H3, Developmental Studies Hybridoma Bank) immunohistochemistry was performed using a peroxidase-conjugated goat anti-rabbit (Pierce) secondary antibody. Fluorescence immunolabelling was performed on transverse cryosections (14 µM) of E9.5 and E10.5 paraformaldehyde-fixed embryos, with p-ERK1/2 and FGF2 primary antibodies (refs. as above) revealed with Alexa 555 secondary antibody, and nuclei stained with 4′,6-diamidino-2-phenylindole (DAPI). Procedures for conventional histology can be found at http://www.empress.har.mrc.ac.uk/browser/, Histology section. Four to ten *Raldh2^−/−^* embryos were examined for all assays and at each developmental stage described hereafter. All spinal cord sections displayed are at the lower cervical/brachial level.

### Embryonic spinal cord derived neurosphere culture

The dorsal region of the brachial/cervical spinal cord was dissected from individual E12.5 embryos. Neurosphere experiments were performed as described [111] with slight modifications. Briefly, cells from 4 spinal cord tissue explants were dissociated in 500 µL of non-enzymatic cell dissociation medium (Sigma) with a fired-polished Pasteur pipette. Cells were plated in 6-well plates (Nunc, non-coated) at a density of 60,000 cells/mL in DMEM:F12 medium (Invitrogen) supplemented with 40 ng/mL FGF2 (human, Peprotech), and 20 ng/mL EGF (mouse, Sigma). Half medium was changed every 2 days. Spheres were counted under a Nikon light microscope and allowed to differentiate on laminin (Gibco)-coated 4 mm coverslips before immunostaining. Immunocytochemisty was performed according to ref. [76]. Anti-β-III-tubulin (TuJ1, Covance) and anti-Nestin (rat-401, Developmental Studies Hybridoma Bank) were used at 1/600 and 1/100 dilutions, respectively. Nuclei were stained with 0.5 µg/mL diamino-2-phenyle-indole (DAPI, Sigma).

### Flow cytometric analysis

Lower cervical/brachial E12.5 spinal cords were dissected, washed in Hanks balanced salt solution (HBSS), and resuspended in a Liberase Blendzyme IV digestion buffer (Roche) solution for 5 minutes at 37°C. A 40-µm filtered supernatant was diluted with an equal volume of 20% serum (FBS) in Dulbecco modified Eagle medium (DMEM, Invitrogen) to stop the reaction, and tissues were pelleted and washed. After resuspension in DMEM containing 2% fetal calf serum (FCS) and 10 mM HEPES, cell concentration was adjusted to 10^6^ cells/mL using DMEM. Hoechst 33342 (Sigma) was added at 5 µg/mL and the cell suspension incubated at 37°C for 60 minutes. For control experiments, the channel blocker verapamil was added at a concentration of 50 µM for 5 minutes prior to the addition of Hoechst 33342. Cell suspensions were mixed every 20 minutes, and resuspended in 4°C HBSS containing 2% FCS and 2 µg/mL propidium iodide to assay for viability. Flow cytometric analysis and fluorescence-activated cell sorting (FACS) was carried out immediately afterward using a Dako triple laser cell sorter (MoFlow, Cytomation, Fort Collins, CO). For detailed descriptions of Hoechst 33342 staining and flow cytometry, see http://www.bcm.edu/labs/goodell/index.cfm.

### Real-time quantitative RT-PCR

Total RNA (100 ng) was subjected to real-time RT-PCR using SYBR Green Core Reagents (Qiagen) according to the manufacturer's protocol. The incorporation of SYBR Green dye into the PCR products was monitored in real-time with an ABI PRISM 7700 sequence detection system (PE Applied Biosystems). Target genes were quantified relative to a reference gene, glyceraldehyde-3-phosphate dehydrogenase (*Gapdh*), which expression was stable in our experimental conditions. Primer sequences are available on request.

### Microarray analysis

Total RNA (20 µg+) was extracted from dissected brachial spinal cords from ten WT and *Raldh2^−/−^* mutants collected at E12.5 after short-term RA rescue. RNA was purified using the RNeasy kit (Qiagen). An Agilent low RNA input linear amplification kit was used on sorted SP populations. RNA quality was verified by using an Agilent 2100 Bioanalyzer. All subsequent reactions using Affymetrix GeneChip Mouse 430A arrays were carried out by the Baylor College of Medicine Microarray Facility as indicated at http://www.bcm.edu/mcfweb/, and in compliance to MIAME guidelines. Data normalization and statistical analysis was performed using dChip 1.2 (W. Wong, Harvard University, Cambridge, MA). Genes were classified into functional categories using the EASE analysis tool (http://david.abcc.ncifcrf.gov/) based on the classification scheme in Gene Ontology.

## Supporting Information

Figure S1 **Lack of RALDH2-mediated RA synthesis affects early neuronal determinants in the prospective spinal cord.** Whole-mount ISH analysis of *Pax6* (A,B), *Ngn2* (C,D), *Olig2* (E,F, main panels) and *Irx3* (E,F, insets) in WT and unrescued *Raldh2^−/−^* embryos (genotypes as indicated in each panel). The developmental stages are 6–8 somites (A–D, insets in E,F) and E9.0 (E,F, inset in C,D). fb, forebrain; hb, hindbrain; pl, placodal cells; sc, spinal cord.
(TIF)

Figure S2 **Altered FGF signaling in the developing spinal cord of RA-deficient mutants.** A: Western blot analysis of p-ERK levels in WT and *Raldh2^−/−^* samples. Upper panel: E9.0 (14–16 somite-stage) embryos, caudal regions, pooled, n = 15. Lower panel: E12.5 embryos collected after short-term rescue, upper cervical/brachial level spinal cords, pooled, n = 7. B-E: Whole-mount immunodetection of phosphorylated ERK1/2 (p-ERK1/2) in E8.5 (somitic stages and genotypes as indicatd) WT and *Raldh2^−/−^* embryos. B,C: Profile views; D,E: details of the caudal region viewed dorsally. Brackets in B,C indicate strongly labelled extra-embryonic membranes, and in D separate domains of high labelling in somitic and caudal regions. F–I: Whole-mount in situ hybridization (ISH) analysis of *Spry1* (F,G) and *Fgf18* (H,I) in WT and *Raldh2^−/−^* embryos (genotypes as indicated). All embryos are viewed dorsally, and the developmental stages are 6–8 somites (F,G, and H,I, insets), and 12–14 somites (H,I, main panels).
(TIF)

Figure S3 **Immunofluorescence analysis of FGF2 and pERK1/2 distribution in the neural tube of E9.5 unrescued embryos (A–D), and E10.5 embryos after short-term RA-rescue (E,F).** Transverse sections at cervico-brachial levels. Views of the immunofluorescence and of merged images with DAPI staining are shown side by side (right and left panels, respectively, with embryo genotypes indicated in the merged images). Brackets (A,B) highlight the ventricular cell layer in the dorsal neural tube.
(TIF)
