# Supplementary figures and images for: Retinoic Acid-Dependent Signaling Pathways and Lineage Events in the Developing Mouse Spinal Cord
Source: PLoS One. 2012 Mar 2;7(3):e32447. doi: 10.1371/journal.pone.0032447 (PMC3292566; doi:10.1371/journal.pone.0032447)

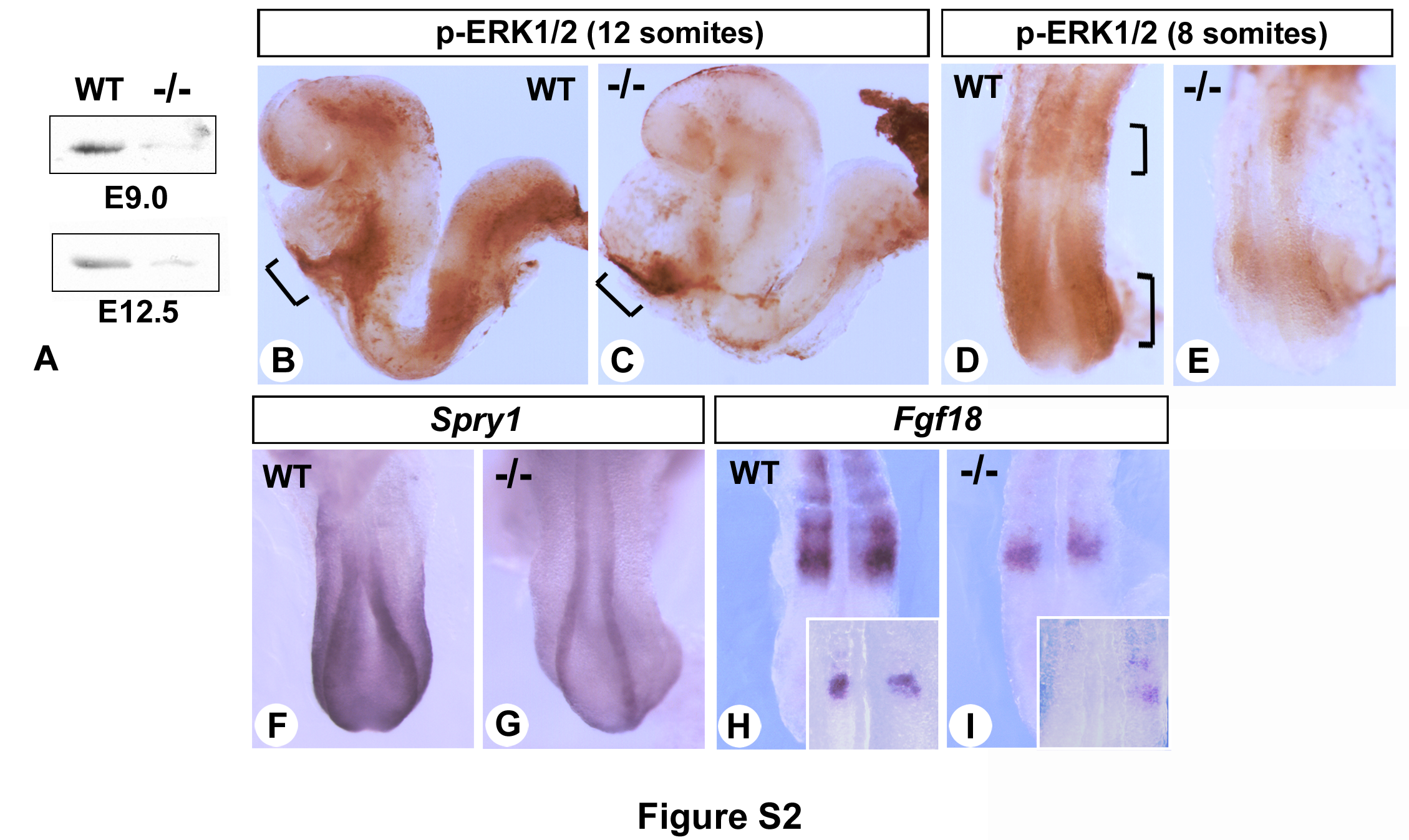

Supplement: Figure S2 — Altered FGF signaling in the developing spinal cord of RA-deficient mutants. A: Western blot analysis of p-ERK levels in WT and Raldh2−/− samples. Upper panel: E9.0 (14–16 somite-stage) embryos, caudal regions, pooled, n = 15. Lower panel: E12.5 embryos collected after short-term rescue, upper cervical/brachial level spinal cords, pooled, n = 7. B-E: Whole-mount immunodetection of phosphorylated ERK1/2 (p-ERK1/2) in E8.5 (somitic stages and genotypes as indicatd) WT and Raldh2−/− embryos. B,C: Profile views; D,E: details of the caudal region viewed dorsally. Brackets in B,C indicate strongly labelled extra-embryonic membranes, and in D separate domains of high labelling in somitic and caudal regions. F–I: Whole-mount in situ hybridization (ISH) analysis of Spry1 (F,G) and Fgf18 (H,I) in WT and Raldh2−/− embryos (genotypes as indicated). All embryos are viewed dorsally, and the developmental stages are 6–8 somites (F,G, and H,I, insets), and 12–14 somites (H,I, main panels). (TIF) [file pone.0032447.s002.tif]

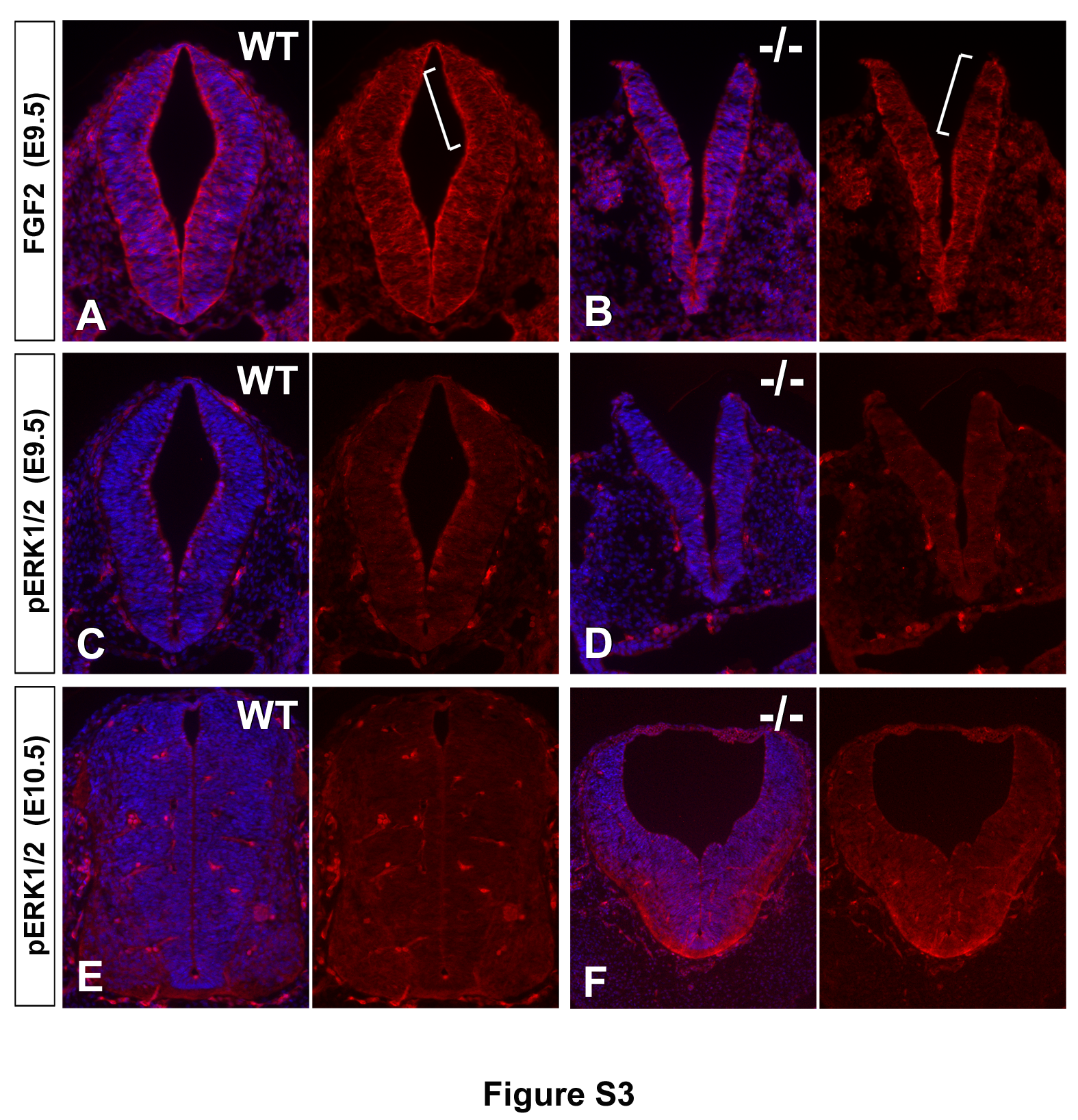

Supplement: Figure S3 — Immunofluorescence analysis of FGF2 and pERK1/2 distribution in the neural tube of E9.5 unrescued embryos (A–D), and E10.5 embryos after short-term RA-rescue (E,F). Transverse sections at cervico-brachial levels. Views of the immunofluorescence and of merged images with DAPI staining are shown side by side (right and left panels, respectively, with embryo genotypes indicated in the merged images). Brackets (A,B) highlight the ventricular cell layer in the dorsal neural tube. (TIF) [file pone.0032447.s003.tif]
